# The Royal Visit to the London Hospital

**Published:** 1903-06-20

**Authors:** 


### THE ROYAL VISIT TO THE LONDON HOSPITAL.

THE visit of the King and Queen to the London Hospital on the 11th instant, to open the new building, was in all respects, except the weather, a remarkable success, and the arrangements were admirable. Their Majesties were received by the Duke of Cambridge, president of the Hospital; the Hon. Sydney Holland, chairman; and other officials. In reply to the address which was read by Mr. Holland, the King said:

"It gives myself and Queen Alexandra great pleasure to be here to-day and to open this building. It is a great satisfaction to me to see such a great advance in hospital construction and policy, and I am glad to think that the out-patients in this hospital will be treated with greater care and attention than were possible in your old building. I am unable to thank the generous donor who has given £25,000 to this building; but it must be a pleasure to him to see his gift so well bestowed; and I am specially glad to learn that the benefits of the London Hospital are open to anyone who is poor an dailing without having previously to wait to obtain a letter from some subscriber. (Cheers.) I note also with satisfaction the help the hospital has received from the great employers in the nighbourhood; and looking at the report I notice as a matter of satisfaction that your treasurer, Mr. John Henry Buxton, succeeds his father as treasurer and has also a son on the committee. I note also the very generous help the hospital has received, especially from the Lord Mayor and several great societies. You have rightly alluded to the interesting fact that the Duke of Cambridge has presided over this hospital for 50 years. (Loud cheers.) We are all delighted to see him here to-day, and trust he may long be spared to continue his duties as president. I well remember the visit of Queen Victoria, my beloved mother, and I know of the great interest she took in this hospital. Speaking for Queen Alexandra, I can say that she is very much pleased with the way in which this hospital has carried out the trust she imposed on it; and it is a very great satisfaction to me that she has been able to introduce into this country from her own country the invention of her distinguished countryman, Dr. Finsen, for the treatment of lupus, which has already produced such satisfactory results. We shall have great satisfaction in inspecting the building; and before declaring it open I wish to express my deep feeling of gratitude to this hospital, which, at the time of my severe illness, provided me with such a distinguished surgeon as Sir Frederick Treeves (loud cheers), with an anæsthetist in Dr. Hewitt, and two such nurses—Nurse Haines and Nurse Tarr (prolonged cheers)—whose unceasing attention I cannot too highly praise. I declare this building open, and may God's blessing rest on all those who come to it and on all those who work in it."

There was also a ceremonial in connection with the opening of the extension of the Finsen-light department, and the Queen said: "I am glad indeed that my countryman Dr. Finsen's discovery is so successful, and I am pleased to know that the trust I put upon this hospital has been so well carried out. I thank the patients for their touching mark of gratitude, and hope they will all soon be restored to health. I declare these rooms open."

THE Worshipful Company of Skinners have made a grant of five guineas to the After-Care Association for Poor Persons Discharged Recovered from Asylums for the Insane.

